# Clinical and Renal Biopsy Findings Predicting Outcome in Renal Thrombotic Microangiopathy: A Large Cohort Study from a Single Institute in China

**DOI:** 10.1155/2014/680502

**Published:** 2014-08-11

**Authors:** Xiao-Juan YU, Feng YU, Di Song, Su-Xia Wang, Yan Song, Gang Liu, Ming-Hui Zhao

**Affiliations:** ^1^Renal Division, Department of Medicine, Peking University First Hospital, Institute of Nephrology, Peking University, Key Laboratory of Renal Disease, Ministry of Health of China, Key Laboratory of CKD Prevention and Treatment, Ministry of Education of China, Beijing 100034, China; ^2^Department of Nephrology, The First Affiliated Hospital of Chinese PLA General Hospital, 51 Fucheng Road, Beijing 100048, China; ^3^Peking-Tsinghua Center for Life Sciences, Beijing 100871, China

## Abstract

*Objective*. The current study aimed to investigate the spectrum of etiologies and associated disorders of renal biopsy-proven thrombotic microangiopathy (TMA) patients. *Methods*. The clinical, laboratory, and renal histopathological data of patients with renal TMA from 2000 to 2012 in our institute were collected and reviewed. *Results*. One hundred and nine TMA patients were enrolled in this study. The mean age was 34.0 ± 11.1 years. Seventy patients (64.2%) were male and thirty-nine patients (35.8%) were female. There were eight patients (7.3%) with hemolytic uremic syndrome (HUS). Sixty-one patients (56.0%) were secondary to malignant hypertension. Fourteen patients (12.8%) were pregnancy-associated TMA. Other associated disorders included 17 patients with connective tissue disorders, 2 patients with hematopoietic stem cell transplantation, 4 patients with Castleman's disease, 1 patient with cryoglobulinemia, and 2 patients with glomerulopathy. During followup, 8 patients died due to severe infection, 17 patients had doubling of serum creatinine, and 44 had end-stage renal disease. In multivariate analysis, male, elevated serum creatinine, and decreased hemoglobin were independently associated with poor renal outcomes. *Conclusions*. Renal TMA changes consisted of different disorders with various etiologies. aHUS, pregnancy-associated TMA, and malignant hypertension accounted for the majority of patients in our cohort.

## 1. Introduction

Thrombotic microangiopathy (TMA) was defined as the pathological lesions characterized by endothelial injury and platelet-rich thrombi formation in microvasculature, leading to microangiopathic hemolytic anemia, consumptive thrombocytopenia, and end-organ ischemic damages. It consisted of a group of different diseases. Hemolytic uremic syndrome (HUS) due to either Shiga toxin-producing bacteria (D + HUS), invasive* Streptococcus pneumoniae* infection (p-HUS), or complement alternative pathway dysregulation (atypical HUS, aHUS) and thrombotic thrombocytopenic purpura (TTP) represent the most classic TMA. TMA may also occur in other related conditions, such as malignant hypertension, pregnancy, connective tissue disorders, hematopoietic stem cell transplantation, malignancy, drug toxicity, and glomerulopathy [1]. As TMA represents a histological description with endothelial injury resulting from various causes as the hallmark change, different strategies of treatment and variable prognosis were often observed. TMA-associated pathologic changes can cause multiple organ lesions leading to a variety of clinical manifestations, in which kidney is the most commonly affected organ, and renal involvement is associated with poor outcome [2–9]. However, detailed descriptions of renal injury features, especially pathological indices and long-term outcomes, are lacking for decades.

In the current retrospective study, we aimed to investigate the spectrum of etiologies and associated disorders of 109 renal biopsy-proven TMA patients in our institute. The associations between clinical, laboratory, and pathological characteristics and outcomes were further analyzed.

## 2. Methods

### 2.1. Patients

Clinical and renal histopathological data of patients with renal biopsy-proven thrombotic microangiopathy, diagnosed between January 2000 and December 2012 in Peking University First Hospital, were reviewed. Kidney injury is usually minimal or absent in TTP and patients with TTP often presents with more severe thrombocytopenia than HUS. As a result, renal biopsy is seldom done in TTP, contributing to the rarity of TTP in our institute. In order to minimize the bias of patients recruitment, TTP was excluded in our study.

The clinical diagnosis of TMA was characterized by microangiopathic hemolytic anemia and thrombocytopenia and/or fever and/or acute renal impairment and/or neurologic impairment [1, 4, 5, 7]. The pathological diagnosis of renal TMA was defined by the following: in acute phase, it was marked with luminal narrowing or total occlusion by intraluminal, subendothelial, or medial accumulation of eosinophilic, fuchsinophilic material with staining properties of fibrin, invariably associated with endothelial swelling, denudation, and sometimes fragmented and/or hemolyzed erythrocytes. In chronic phase, mucoid edema of the intima and/or the “onion skin” type of intimal fibroplasia may occur [10, 11].

Informed consent was obtained from each patient for blood sampling and renal biopsy. The research was in compliance with the Declaration of Helsinki and was approved by the local ethical committees.

### 2.2. Clinical Assessment

**Figure 1:**
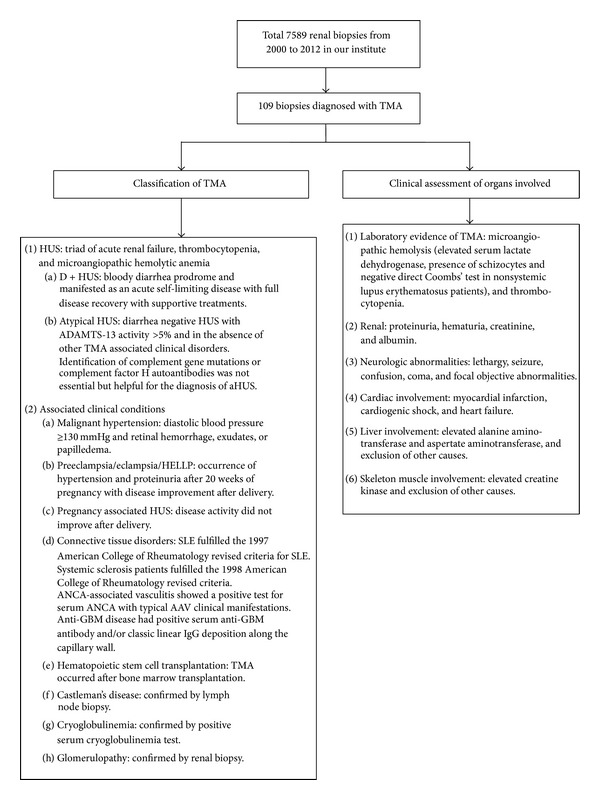
Classification and clinical assessment of patients with TMA.

The TMA patients were classified according to etiologies or associated clinical conditions. Clinical data including organs involved were further collected (as shown in Figure 1).

#### 2.2.1. Scoring System of the Clinical and Laboratory Parameters in TMA

The clinical disease activity of TMA patients was assessed by the Rose scoring system, which was modified by Wyllie BF with the following features: neurological findings (absent, present), serum creatinine value (<132 *μ*mol/L, 132~221 *μ*mol/L, and >221 *μ*mol/L), platelet count (>100 × 10^9^/L, 20~100 × 10^9^/L, and <20 × 10^9^/L), and hemoglobin level (>12.0 g/dL, 9.0~12.0 g/dL, and <9.0 g/dL). The final scores ranged from 0 to 7 [12, 13].

The response to therapy includes complete remission, partial remission, and treatment failure [14, 15]. Complete remission was defined as normalization of hematologic values (a normal platelet count and lactate dehydrogenase level), clinical improvement of all affected organs, and no clinical worsening in any vital organ. Partial remission was defined as hematologic improvement and no clinical worsening in any vital organ. Treatment failure was defined as no improvement of hematologic values or clinical worsening of any vital organ.

The patients were followed up in our outpatient clinic. The primary end point was defined as death, and the secondary end point was defined as end stage renal disease (ESRD) or doubling of serum creatinine.

### 2.3. Laboratory Assessment

Complete blood count, plasma lactate dehydrogenase, peripheral blood smear, urinalysis, serum creatinine, liver enzymes, cardiac troponin I, and creatine kinase were collected before treatment. Plasma C3 and C4 levels were measured using rate nephelometric immunoassays (Beckman-Coulter, IMMAGE, USA).

The serum ADAMTS-13 activity was assessed using a residual collagen-binding assay as previously reported [16]. Detection of serum CFH was the same as previously described [17]. Serum CFH autoantibodies were detected by ELISA as described previously [17, 18].

### 2.4. Renal Histopathology

Renal biopsy specimens were examined by direct immunofluorescence, light microscopy, and electron microscopy. Two pathologists evaluated the biopsies separately, blinded to patients' data and scores of other observers. Differences in scoring between the pathologists were resolved by rereviewing the biopsies and thus reaching a consensus. Patients with <10 glomeruli and <6 vessels in renal biopsies were excluded.

#### 2.4.1. Direct Immunofluorescence Examination

Fresh frozen tissue sections were stained immediately after the renal biopsy with fluorescein isothiocyanate- (FITC-) labelled rabbit anti-human IgG, IgA, IgM, C3c, C1q, and fibrin antibodies (DAKO A/S, Copenhagen, Denmark). Results were graded from 0 to 4 according to the intensity of fluorescence.

#### 2.4.2. Light Microscopy Examination

Renal biopsy specimens were fixed in 4.5% buffered formaldehyde for light microscopy. Consecutive serial 3 *μ*m sections were used for histological staining. Sections were stained with haematoxylin and eosin, periodic acid-Schiff, periodic acid-silver methenamine, and Masson's trichrome.

### 2.5. Electron Microscopy Examination

Renal biopsy materials were fixed in 2.5% paraformaldehyde for electron microscopy. After being embedded in epon, ultrathin sections were mounted on metal grids and stained with uranyl acetate before being viewed in a transmission electron microscope (JEM-1230; JEOL, Tokyo, Japan).

#### 2.5.1. Quantification of Pathological Morphology in Renal TMA

The renal TMA lesions were scored according to the previous reports [9, 19]. The lesions were divided into acute changes and chronic changes. The acute lesion was defined as the presence of at least 1 fibrin microthrombus, either in glomeruli or in small arterioles and/or in arteries; the presence of acute lesion was scored as 1 and the absence as 0. Chronic changes were mucoid changes and onion skin lesions of arterioles and/or arteries. Presence of chronic changes was scored as 1 and absence as 0.

The renal TMA pathologic changes were classified as predominant involvement of glomeruli or artery/arteriole or equal involvement of glomeruli and artery/arteriole.

### 2.6. Statistical Analysis

Statistical software SPSS 13.0 (SPSS, Chicago, IL, USA) was used for statistical analysis. Quantitative data were expressed as mean ± sd for normally distributed data or median with 25% and 75% interquartile range for nonnormally distributed data. Differences between groups of normally distributed and homogeneous quantitative data were tested with One-Way ANOVA, and Kruskal-Wallis* H* test was used for nonnormally distributed or heterogeneous quantitative data. Polychotomous data were compared using the chi-square or Fisher's exact test. Differences of semiquantitative data were tested with the Kruskal-Wallis* H* test and the Mann-Whitney* U*-test. Kaplan-Meier curves were used to analyze patients' prognosis. Univariate survival analysis was carried out using the log-rank test. Multivariate analysis of patient survival was performed using the Cox regression model. The following variables were assessed as potential predictors of death and renal outcomes: age, sex, serum creatinine score (<132 *μ*mol/L: score 0; 132~221 *μ*mol/L: score 1; >221 *μ*mol/L: score 2), proteinuria, plasma albumin level, hematuria, hemoglobin score (>12.0 g/dL: score 0; 9.0~12.0 g/dL: score 1; <9.0 g/dL: score 2), platelet score (>100 × 10^9^/L: score 0; 20~100 × 10^9^/L: score 1; <20 × 10^9^/L: score 2), neurological involvement, modified Rose score, acute renal TMA pathologic changes, chronic renal TMA pathologic changes, and location of TMA pathologic changes. Variables that did not affect survival significantly were removed by a stepwise procedure according to a likelihood ratio test. Results were expressed as hazard ratio with 95% confidence intervals. Statistical significance was considered as *P* < 0.05.

## 3. Results

One hundred and nine TMA patients were included in this study, who accounted for 1.4% (109/7589) of the total biopsied cases at the same period in our nephrology center. The mean age was 34.0 ± 11.1 years at presentation. Seventy patients (64.2%) were male and thirty-nine patients (35.8%) were female.

### 3.1. General Classification of TMA Patients

The classification of the 109 TMA patients based on etiologies and associated clinical conditions was listed in Table 1. HUS accounted for 7.3% (1 patient with D + HUS and 7 patients with aHUS). Sixty-one patients (56.0%) were secondary to malignant hypertension as the following: 32 (29.4%) with primary malignant hypertension and 29 (26.6%) with secondary malignant hypertension including 19 with IgA nephropathy, 5 with primary mesangial proliferative glomerulonephritis, 4 with hyperaldosteronism, and 1 with renal artery stenosis. Fourteen patients (12.8%) were pregnancy-associated TMA. Other TMA associated disorders included 17 patients with connective tissue disorders (12 patients with systemic lupus erythematosus (SLE), 3 patients with systemic sclerosis, 1 patient with antineutrophil cytoplasmic autoantibody- (ANCA-) associated vasculitis, and 1 patient with anti-glomerular basement membrane (GBM) associated disease); 2 patients associated with hematopoietic stem cell transplantation, 4 patients with Castleman's disease; 1 patient with cryoglobulinemia. Two patients were associated with glomerulopathy including 1 with hepatitis B virus-associated membranous nephropathy and 1 with IgA nephropathy without clinical and pathological malignant hypertension manifestations.

### 3.2. Clinical, Laboratory, and Renal Pathological Data of Patients with TMA

Of all the 109 TMA patients, fever was recorded in 12 (11.0%) patients. Five (4.6%) patients presented with evidence of neurologic involvement, nine (8.3%) patients with liver injury, 1 patient (0.9%) with cardiac involvement, and 1 patient (0.9%) with skeleton muscle involvement.

The average levels of hemoglobin, platelet count, lactate dehydrogenase, and schistocytes on peripheral blood smears were 95.1 ± 30.0 g/L, 165 ± 95 × 10^9^/L, 264 IU/L (182 IU/L, 472 IU/L), and 0.1% (0%, 0.2%), respectively. Microscopic hematuria could be seen in 70 (64.2%) patients. The median level of proteinuria was 2.90 g/d (1.35 g/d, 5.42 g/d), and nephrotic range proteinuria was seen in 42 patients (38.5%). The median serum creatinine value was 478.00 *μ*mol/L (234.50 umol/L, 768.50 *μ*mol/L) with 96 patients (88.1%) who had elevated serum creatinine at diagnosis. The mean modified Rose score was 3.2 ± 1.6.

On renal biopsy examinations, acute TMA changes were seen in 26 (23.9%) patients, and chronic TMA changes were seen in 75 (68.8%) patients. Thirteen (11.9%) patients showed renal TMA pathologic changes in glomeruli, 79 (72.5%) in artery/arteriole, and 17 (15.6%) in both sites.

Fifteen (13.8%) patients received plasma exchange or plasma infusion therapy. Forty-eight (44.0%) patients were treated with corticosteroid and 28 (25.7%) with other immunosuppressants (details in Table 2).

The mean follow-up time was 30.5 ± 36.5 months. Forty-one patients (37.6%) had complete remission, 3 patients (2.8%) had partial remission, and 65 patients (59.6%) had treatment failure at the end of followup. Eight patients (7.3%) died due to severe infection. With regard to the long-term renal outcomes, 61 patients (56.0%) reached the secondary end point, including 17 patients with doubling of serum creatinine and 44 patients with end-stage renal disease (details in Table 3).

In univariate survival analysis, we did not find risk factor indicating death. Male, elevated serum creatinine value, decreased hemoglobin level, higher modified Rose score, presence of chronic renal TMA pathologic changes, and isolated artery/arteriole TMA pathologic changes were observed to be associated with poorer renal outcomes (Table 4). In a further multivariate Cox hazard analysis, being a male, elevated serum creatinine, and decreased hemoglobin level were identified as independent risk factors for long-term renal outcomes (Table 5).

### 3.3. Comparisons of Clinical and Renal Pathological Features of Patients with aHUS, Pregnancy-Associated TMA, and Malignant Hypertension

As the patients with aHUS, pregnancy-associated TMA, and malignant hypertension accounted for the majority of total TMA cases, we further analyzed the clinical and renal pathological differences among the 3 groups: group 1, aHUS; group 2, pregnancy-associated TMA; group 3, malignant hypertension.

The comparisons of clinical, laboratory, and renal pathological data among the three groups were shown in Table 6. Patients with pregnancy-associated TMA had higher ratio of liver involvement than that in the other 2 groups. Patients with aHUS had the highest serum creatinine value and the lowest hemoglobin value compared with the other 2 groups. Patients with aHUS and pregnancy-associated TMA had lower platelet count than that in malignant hypertension group. Patients in pregnancy-associated TMA group had the highest lactate dehydrogenase level compared to the other two groups. In total, patients with aHUS had the highest modified Rose scores in comparison with the other 2 groups.

Patients with aHUS presented with the highest prevalence of acute renal TMA pathologic changes and patients with malignant hypertension had the highest ratio of chronic renal TMA pathologic changes. Renal TMA pathologic changes were commonly present in the arteries or arterioles in malignant hypertension, and in the other 2 groups, it could be seen in both glomeruli and arteries/arterioles.

**Figure 2:**
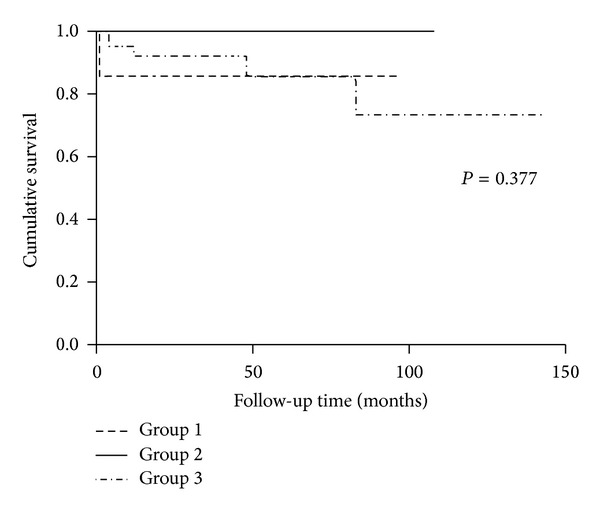
Survival analysis of patients with aHUS, pregnancy-associated TMA, and malignant hypertension. (Group 1: aHUS. Group 2: pregnancy-associated TMA. Group 3: malignant hypertension).

**Figure 3:**
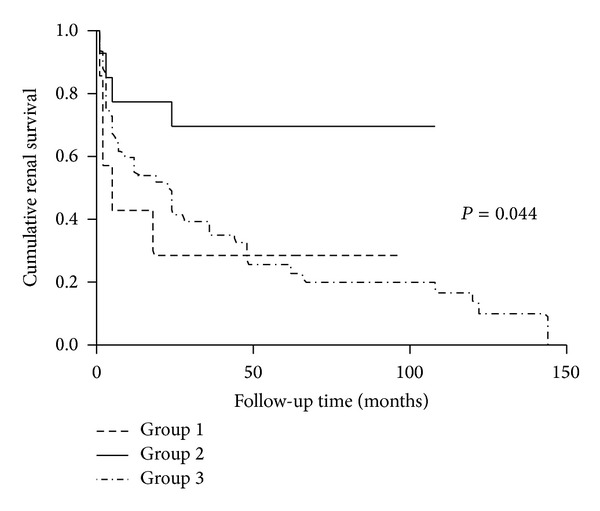
Renal survival analysis of patients with aHUS, pregnancy-associated TMA, and malignant hypertension. (Group 1: aHUS. Group 2: pregnancy-associated TMA. Group 3: malignant hypertension).

Regarding long-term endpoints, there was no significant difference for patients' survival among the three groups (*P* = 0.377, Figure 2). However, patients with aHUS and malignant hypertension presented with poorer renal outcomes than that in pregnancy-associated TMA group. (*P* = 0.044, Figure 3).

## 4. Discussion

TMA is a group of systemic disorder with abnormalities in multiple organs. Most patients presented with renal involvement, which was associated with high frequencies of mortality and ESRD [2–9]. Here we described the clinical and pathological features of a large cohort of Chinese renal TMA patients.

In our center, adult renal TMA accounted for 1.4% of the total biopsied cases at the same period, which showed that it was not uncommon. Idiopathic TMA, including D + HUS and aHUS, only accounted for 7.3% of the total TMA cases, and the majority of the patients were secondary TMA. The spectrum was significantly different from other reports, such as in the Oklahoma TTP-HUS Registry from 1995 to 2009; idiopathic TTP-HUS plus bloody diarrhea prodromal HUS accounted for 46.6% of all the 283 patients [20], and in Japan Nara Medical University TMA registry from 1998 to 2008, congenital and idiopathic TTP-HUS plus* E. coli* O157:H7-associated TMA consisted of 60.1% of the 919 TMA patients [21]. The reasons for the intercenter discrepancy might be attributed to the following. (1) As HUS typically occurs in infancy and childhood [1, 22] and patients enrolled in our cohort were mainly adults, this biased recruitment might be associated with the rarity of HUS in our study. (2) From previous reports, aHUS was nearly 1/3 to 1/2 as frequent as TTP [23]. Kidney injury is usually minimal or absent in TTP and patients with TTP often presents with more severe thrombocytopenia than HUS. As a result, renal biopsy is seldom done in TTP, contributing to the rarity of TTP in our institute. In order to minimize the bias of patients recruitment, TTP was excluded in our study. (3) TMA due to malignant hypertension was the most common clinical disorder (56.0%) in our study, which was significantly different from other centers, which only accounted for 2.1% of all the TMA patients from Oklahoma Registry data. Importantly, the latter registry was mainly based on data of patients who received plasma exchange [24], and plasma treatment was not the common choice of therapy for malignant hypertension.

TMA affects multiple organs and kidney is the most commonly involved organ. As the enrollment of 109 patients in our study was based on the renal biopsy database, we had a chance to further analyze renal clinical, laboratory, pathological characteristics and outcomes which were absent in the literatures. We found that most of the patients presented with more severe renal function damage, such as serum creatinine value and the ratio of acute renal failure, than that in the previous report [25]. Proteinuria was seen in all the patients and 64.2% of patients had hematuria. The renal biopsy results showed different degrees of acute and/or chronic TMA changes in glomeruli and/or arteries/arterioles. The overall prognosis was unsatisfactory with 7.3% of patients dying and 56.0% of patients reaching secondary endpoints, including ESRD or doubling of serum creatinine. In multivariate analysis, we found that male, elevated serum creatinine value, and decreased hemoglobin level were independent risk factors for poorer renal outcomes. As it was a retrospective study within 12 years, the high rate of treatment failure (59.6%), attributed to inappropriate therapy, such as low rate of plasma exchange and lack of anticomplement therapy, might also contribute to the poor prognosis. Importantly, in the past decades, the outcomes of TMA have significantly improved based on the fast diagnosis and therapeutic guidelines [26, 27] with more clear understandings of its pathogenesis and new treatment agents.

As the patients with aHUS, pregnancy-associated TMA, and malignant hypertension accounted for the majority of total TMA cases in our cohort, we further analyzed the clinical and renal pathological differences among the 3 groups. We found that patients with aHUS had more severe TMA injury features, including higher modified Rose score, higher serum creatinine value, higher ratio of acute renal TMA pathologic changes, lower hemoglobin value, and lower platelet count than those in the other 2 groups. However, patients with malignant hypertension had higher prevalence of chronic renal TMA pathologic changes, including arteries and/or arterioles mucoid changes and onion skin-like lesions. These might contribute to those patients with aHUS and malignant hypertension presenting with poorer renal outcomes than that in pregnancy group. In fact, as the pregnancy-associated TMA consisted of different spectrums of diseases with different pathogeneses, such as preeclampsia/eclampsia/HELLP due to aberrant placental vasculature development and function, leading to the release of placenta-derived anti-angiogenic factors soluble fms-like tyrosine kinase 1 (sFlt-1), soluble endoglin, and so forth [15, 28, 29] and pregnancy-associated TTP-HUS similar to TTP and HUS, including ADAMTS-13 deficiency and complement dysregulation, in which case pregnancy was more like a trigger for the development of TMA in some patients [30, 31], thus, the heterogeneity of this group needs further detailed explorations with larger samples.

There were some limitations in our study. As this was a retrospective study, some laboratory examinations, such as serum ADAMTS-13 activity, shiga toxin, and complete complement assessment, were not available in all patients. Malignant hypertension especially might be a feature of TMA, and some patients, included in “malignant hypertension group” presenting with poor renal outcomes in our study, might have other reasons of TMA as they only received supportive treatment. This needs to be further explored.

In conclusion, renal TMA changes consisted of different disorders with various etiologies, and the patients with aHUS, pregnancy-associated TMA, and malignant hypertension accounted for the majority of total TMA numbers in our cohort. The explorations of more clear understandings of its pathogenesis and standard treatment guidelines are needed.

## Figures and Tables

**Table 1:** Classification of patients with thrombotic microangiopathy.

Etiologies or associated disorders	Number of patients (%)
Total	109
Idiopathic	
D + HUS	1 (0.9)
aHUS	7 (6.4)
Secondary	
MHT	61 (56.0)
Pregnancy associated	
Preeclampsia/HELLP	5 (4.6)
HUS	9 (8.3)
Others	
Connective tissue disorders	17 (15.6)
HSCT	2 (1.8)
Castleman's disease	4 (3.7)
Cryoglobulinemia	1 (0.9)
Glomerulopathy	2 (1.8)

Notes: D + HUS: diarrhea positive hemolytic uremic syndrome; aHUS: atypical hemolytic uremic syndrome; MHT: malignant hypertension; HELLP: hemolysis, elevated live enzymes, low platelet count; HSCT: hematopoietic stem cell transplantation.

**Table 2:** Treatment of patients with thrombotic microangiopathy.

Etiologies or associated disorders (109 patients in total)	Treatment			
	PI/PE	CS	Cytotoxic agents	ACEI/ARB
D + HUS (*n* = 1)	0	0	0	1
aHUS (*n* = 7)	4	6	3 (CYC)	5
MHT (*n* = 61)	0	10	6 (CYC or MMF)	40
Pregnancy associated				
Preeclampsia/HELLP (*n* = 5)	2	2	0	3
HUS (*n* = 9)	5	6	2 (CYC)	4
Others				
Connective tissue disorders (*n* = 17)	4	17	12 (CYC or LEF)	10
HSCT (*n* = 2)	0	2	1 (CsA)	0
Castleman's disease (*n* = 4)	0	3	2 (chemotherapy)	1
Cryoglobulinemia (*n* = 1)	0	1	1 (LEF)	1
Glomerulopathy (*n* = 2)	0	1	1 (CYC)	1

Notes: D + HUS: diarrhea positive hemolytic uremic syndrome; aHUS: atypical hemolytic uremic syndrome; MHT: malignant hypertension; HELLP: hemolysis, elevated live enzymes, low platelet count; HSCT: hematopoietic stem cell transplantation; PI: plasma infusion; PE: plasma exchange; CS: corticosteroid; ACEI: angiotensin-converting enzyme inhibitors; ARB: angiotensin II receptor blockers; CYC: cyclophosphamide; MMF: mycophenolate mofetil; LEF: leflunomide; CsA: cyclosporine A.

**Table 3:** Prognosis of patients with thrombotic microangiopathy.

Etiologies or associated disorders (109 patients in total)	CR (%)	PR (%)	TF (%)	Primary end point (%)	Secondary end point	
					Doubling of Cr (%)	ESRD (%)
D + HUS (*n* = 1)	1 (100)	0	0	0	0	0
aHUS (*n* = 7)	2 (28.5)	0	5 (71.5)	1 (14.3)	0	4 (57.1)
MHT (*n* = 61)	12 (19.7)	0	49 (80.3)	5 (8.2)	16 (26.2)	28 (45.9)
Pregnancy						
Preeclampsia/HELLP (*n* = 5)	4 (80.0)	0	1 (20.0)	0	0	1 (20.0)
HUS (*n* = 9)	6 (66.7)	0	3 (33.3)	0	0	3 (33.3)
Others						
Connective tissue disorders (*n* = 17)	11 (64.7)	1 (5.9)	5 (29.4)	1 (5.9)	0	4 (23.5)
HSCT (*n* = 2)	1 (50.0)	1 (50.0)	0	0	0	0
Castleman's disease (*n* = 4)	2 (50.0)	0	2 (50.0)	1 (25.0)	1 (25.0)	0
Cryoglobulinemia (*n* = 1)	1 (100)	0	0	0	0	0
Glomerulopathy (*n* = 2)	1 (50.0)	1 (50.0)	0	0	0	0

Notes: CR: complete remission; PR: partial remission; TF: treatment failure; Cr: creatinine; ESRD: end stage renal disease; D + HUS: diarrhea positive hemolytic uremic syndrome; aHUS: atypical hemolytic uremic syndrome; MHT: malignant hypertension; HELLP: hemolysis, elevated live enzymes, low platelet count; HSCT: hematopoietic stem cell transplantation.

**Table 4:** Univariate renal survival analysis of patients with thrombotic microangiopathy.

Parameters	HR	95% CI		*P* value
		Lower	Upper	
Age (years)	0.982	0.956	1.009	0.196
Gender (female versus male)	0.319	0.169	0.602	<0.001
Serum creatinine score	4.002	1.709	9.371	0.001
Proteinuria (g/24 hr)	0.991	0.920	1.068	0.820
Plasma albumin value (g/L)	0.994	0.952	1.037	0.765
Hematuria	1.327	0.777	2.265	0.301
Hemoglobin score	1.659	1.183	2.326	0.003
Platelet score	0.897	0.530	1.516	0.684
Neurological involvement	2.171	0.783	6.017	0.136
Modified Rose score	1.354	1.134	1.616	0.001
Presence of acute renal TMA pathologic changes	1.175	0.641	2.156	0.602
Presence of chronic renal TMA pathologic changes	2.709	1.327	5.532	0.006
Isolated glomerular TMA pathologic changes	0.232	0.057	0.953	0.043
Isolated artery/arteriole TMA pathologic changes	2.104	1.061	4.173	0.033
Use of ACEI or ARB	1.702	0.978	2.962	0.060

Notes: ACEI: angiotensin-converting enzyme inhibitors; ARB: angiotensin II receptor blockers.

**Table 5:** Multivariate renal survival analysis of patients with thrombotic microangiopathy.

	HR	95% CI		*P* value
		Lower	Upper	
Multivariate Cox hazard analysis				
Age (years)	1.001	0.968	1.034	0.961
Gender (female versus male)	0.239	0.114	0.504	<0.001
Serum creatinine score	2.311	0.768	6.953	0.136
Hemoglobin score	1.594	0.807	3.146	0.179
Modified Rose score	1.085	0.623	1.891	0.772
Presence of chronic renal TMA pathologic changes	1.091	0.320	3.717	0.889
Isolated glomerular TMA pathologic changes	0.478	0.083	2.750	0.408
Isolated artery/arteriole TMA pathologic changes	0.848	0.259	2.780	0.786
Multivariate stepwise Cox hazard analysis				
Gender (female versus male)	0.242	0.123	0.477	<0.001
Serum creatinine score	2.573	1.039	6.375	0.041
Hemoglobin score	1.777	1.199	2.633	0.004

**Table 6:** Comparisons of clinical, laboratory, and renal pathological data among patients with aHUS, pregnancy-associated TMA, and malignant hypertension.

	aHUS	Pregnancy associated TMA	Malignant hypertension	*P* value
Number of patients	7	14	61	

Age (years)	41 ± 16	30 ± 4.4	32 ± 6.9	0.122
Gender (male : female)	5 : 2	0 : 14	55 : 6	<0.001
Fever (%)	0	2 (14.3)	5 (8.2)	0.534
Neurological abnormalities (%)	1 (14.3)	1 (7.1)	2 (3.3)	0.401
Liver involvement (%)	0	7 (50)	0	<0.001
Heart involvement (%)	0	0	1 (1.6)	0.840
Hematuria (%)	5 (71.4)	10 (71.4)	37 (60.7)	0.677
Proteinuria (g/d)	3.30 ± 1.29	3.83 ± 3.21	3.46 ± 2.54	0.816
Albumin (g/L)	34.0 ± 5.1	34.2 ± 6.8	36.4 ± 4.9	0.239
Creatinine (*μ*mol/L)	870.0 (671.0–1200)	370.5 (78.5–602.8)	560.0 (291.6–904.9)	0.021
Hemoglobin (g/L)	60 ± 5.8	84 ± 32	102 ± 29	0.037
Platelet count (×10^9^/L)	116 ± 88	114 ± 92	189 ± 83	0.004
Lactate dehydrogenase (IU/L)	551 (266–1360)	1156 (369–2762)	216 (163–309)	<0.001
Modified Rose score	4.7 ± 0.5	3.6 ± 2.3	3.1 ± 1.2	0.001
Acute renal TMA pathologic changes (%)	5 (71.4)	4 (28.6)	7 (11.5)	<0.001
Chronic renal TMA pathologic changes (%)	5 (71.4)	2 (14.3)	58 (95.1)	<0.001
Location of renal TMA pathologic changes				
G (%)	0	4 (28.6)	0	<0.001
A (%)	2 (28.6)	4 (28.6)	60 (98.4)	
G and A (%)	5 (71.4)	6 (42.9)	1 (1.6)	

Notes: aHUS: atypical hemolytic uremic syndrome; G: glomeruli; A; artery/arteriole; G and A: glomeruli and artery/arteriole.
